# Quantitative Metabolomic Analysis of Urinary Citrulline and Calcitroic Acid in Mice after Exposure to Various Types of Ionizing Radiation

**DOI:** 10.3390/ijms17050782

**Published:** 2016-05-20

**Authors:** Maryam Goudarzi, Siddheshwar Chauthe, Steven J. Strawn, Waylon M. Weber, David J. Brenner, Albert J. Fornace

**Affiliations:** 1 Department of Biochemistry and Molecular & Cellular Biology, Georgetown University, 3970 Reservoir Road NW, Washington, DC 20057, USA; steve.strawn@gmail.com (S.J.S.); af294@georgetown.edu (A.J.F.J.); 2 Lombardi Comprehensive Cancer Center, Georgetown University, 3800 Reservoir Road NW, Washington, DC 20057, USA; siddheshwarchauthe@gmail.com; 3 Lovelace Respiratory Research Institute, 2425 Ridgecrest Dr. SE, Albuquerque, NM 87108, USA; wweber@lrri.org; 4 Center for Radiological Research, Columbia University, 630 West 168th Street, VC11-240, New York, NY 10032, USA; djb3@cumc.columbia.edu

**Keywords:** Internal emitters, X-ray irradiation, quantitative metabolomics, multiple reaction monitoring, calcitroic acid, citrulline, biodosimetry

## Abstract

With the safety of existing nuclear power plants being brought into question after the Fukushima disaster and the increased level of concern over terrorism-sponsored use of improvised nuclear devices, it is more crucial to develop well-defined radiation injury markers in easily accessible biofluids to help emergency-responders with injury assessment during patient triage. Here, we focused on utilizing ultra performance liquid chromatography-tandem mass spectrometry (UPLC-MS/MS) to identify and quantitate the unique changes in the urinary excretion of two metabolite markers, calcitroic acid and citrulline, in mice induced by different forms of irradiation; X-ray irradiation at a low dose rate (LDR) of 3.0 mGy/min and a high dose rate (HDR) of 1.1 Gy/min, and internal exposure to Cesium-137 (^137^Cs) and Strontium-90 (^90^Sr). The multiple reaction monitoring analysis showed that, while exposure to ^137^Cs and ^90^Sr induced a statistically significant and persistent decrease, similar doses of X-ray beam at the HDR had the opposite effect, and the LDR had no effect on the urinary levels of these two metabolites. This suggests that the source of exposure and the dose rate strongly modulate the *in vivo* metabolomic injury responses, which may have utility in clinical biodosimetry assays for the assessment of exposure in an affected population. This study complements our previous investigations into the metabolomic profile of urine from mice internally exposed to ^90^Sr and ^137^Cs and to X-ray beam radiation.

## 1. Introduction

In the current socio-political environment, there is increasing concern for improvised radiologic and nuclear devices. In addition, unplanned nuclear and radiological events are also a source of concern with the recent Fukushima disaster as a reminder that we need better countermeasures. To this end, we have described a workflow for characterization of urinary and serum metabolomics signatures after exposure. While most of the effort in radiation biodosimetry and countermeasures has focused on X-ray irradiation [1,2,3,4,5], our recent work on exposure to environmentally persistent internal emitters, ^90^Sr and ^137^Cs, provided additional insight into their *in vivo* metabolomic responses [6,7,8,9]. The results of these studies indicated that intermediate metabolites in energy metabolism, lipid beta-oxidation, and inflammation signaling are some of the most affected pathways after exposure to these internal emitters [6,7,8,9]. While these results are similar to those seen with X-ray beam irradiation at a low dose rate (LDR) of 0.00309 Gy/min and at a high dose rate (HDR) of 1.1 Gy/min, distinct differences have been observed in each case based on the type of exposure and dose rate. Dose rate is an important concept in radiobiology since low dose rates can show differences in a variety of biologic responses. In addition, dose estimation can be complicated particularly at low dose rates, which vary with time as the isotope is excreted (or decayed). Varying dose rate played an important role in our studies on the effects of internal emitters, ^90^Sr and ^137^Cs, for the calculation of *in vivo* whole body dose, which was averaged at each time-point for the estimation of cumulative dose. Our metabolomic studies into the effects of internal exposure by ^137^Cs in mice showed that ^137^Cs exposure triggered a large-scale increase in inflammatory markers, such as arachidonic acid, which subsided 30 days (d) after exposure suggesting adaptation and recovery. However, in the case of ^90^Sr exposure, there was a global and persistent downregulation of several metabolites after exposure, which may be due to a more uniform localization of the radionuclide to the bones. In the course of these initial studies, we detected two urinary ions, which showed distinct changes after exposure based on exposure type and dose rate in mice; however, due to unavailability of commercial standards and analytical separation approaches at the time, we were unable to conclusively identify and quantitate these two spectral features using our previously reported conventional shotgun workflow [6,7]. Here, we report on the identification and the development of quantitative methods for these two radiation-sensitive urinary ions using chemical standards via tandem quadrupole mass spectrometry. The results of the current study expand our knowledge of radiation exposure metabolite markers and the methods developed here may be used in other studies for monitoring the levels of these two urinary metabolites.

The first of these two urinary ions to be identified and quantitated using multiple reaction monitoring (MRM) analysis was calcitroic acid. Calcitroic acid is the terminal product of deactivation of 1α,25-dihydroxyvitamin D_2_ (1α,25-(OH)_2_D_2_) [10]. Vitamin D_2_ and D_3_ are metabolized in a similar fashion producing a hormonally active form of 1α,25-(OH)_2_D_2_. Recent studies have indicated that kidneys and keratinocytes contain the enzymes required for production of calcitroic acid from 1α,25-(OH)_2_D_2_ [11,12] under a regulatory mechanism to maintain vitamin D homeostasis. Although these enzymes are yet to be characterized, it is known that the isomers of the cytochrome P450 (CYP) can carry out a multi-step catalytic reaction to hydrolyze vitamin D and ultimately produce calcitroic acid from 1α,25-(OH)_2_D_3_ [13]. Arguably, these enzymes could be responsible for most, if not all, of the metabolic steps leading to side chain oxidation and to side cleavage of 1α,25-dihydroxyvitamin D_2_. Despite the important role of vitamin D metabolism and evidence that its metabolic intermediates may attenuate cell death induced by ionizing radiation (IR) exposure [14], there is little information on the IR-induced changes in the various genes, proteins and metabolites pertaining to this pathway. Our recent gene expression study carried out on the blood of mice exposed to ^90^Sr and ^137^Cs showed lower expression of two *Cyp* genes, *Cyp4f18* and *Cyp4v3* while a higher expression of *Cyp4v3* was recorded after HDR exposure [15,16,17]. In addition, CYP4 proteins have been shown to metabolize vitamin D and are important in defense against chemical and environmental stressors [18].

The second urinary ion was identified here as citrulline, which is an alpha-amino acid and is mainly synthesized by small bowel enterocytes [19]. It has been speculated that the citrulline concentration is related to small bowel function [20]. Studies have shown that a decrease in citrulline concentration is associated with bacterial translocation [21], elevated intestinal fatty acid binding protein concentration as seen in damaged enterocytes [22], and in patients with intestinal dysfunction [23]. Citrulline also plays an important role in regulating the immune response during inflammation and stress signaling [24]. The enzyme which is responsible for the production of citrulline is nitric oxide synthase (NOS). From the different isomers of NOS, NOS1 is expressed in endothelial cells and is regulated by Ca^2+^ [25]. Upon exposure to IR, NOS1 activity is stimulated as part of an early stress signaling mechanism induced by IR exposure [26], subsequently leading to changes in the plasma levels of citrulline. Several *in vivo* studies have established changes in the plasma levels of citrulline as a reliable indicator for gastrointestinal (GI) injury and citrulline has even been suggested as a promising biomarker of GI toxicity in patients undergoing chemo- and radiation therapy [26]. We have also observed a slight yet statistically significant increase in the gene expression of *NOS1* in the blood of mice internally exposed to the radionuclide ^90^Sr [15,16].

The results of these gene expression studies along with the unique changes in the urinary excretion of citrulline and calcitroic acid reported here under various exposure senarios further provide proof for the IR responsiveness of these two metabolites and the robustness of their responses post exposure. Thus, these two biomarkers bear the potential of having applications in large-scale biodosimetry approaches and can help with the refinement of radiation exposure monitoring bio-signature. Furthermore, this study serves as an example of how quantitative mass spectrometry-based metabolomics may be reliably employed in developing a high throughput radiation biodosimetry assay with future clinical implications.

## 2. Results

**Figure 1:**
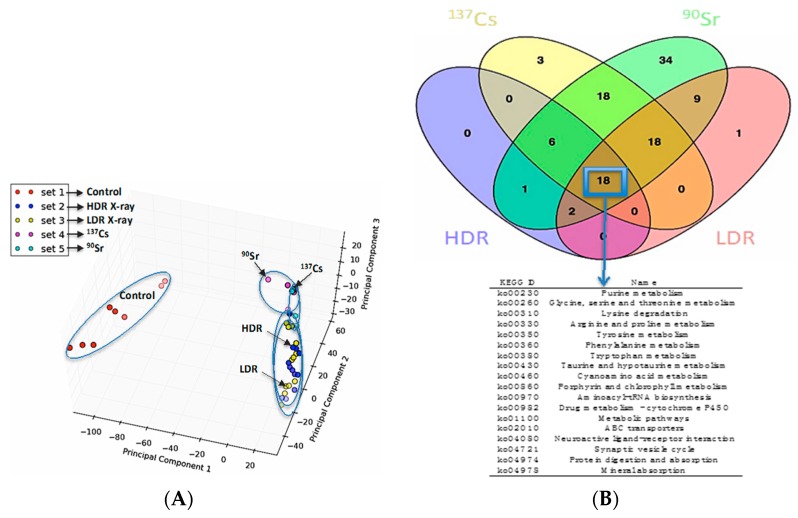
The overall urinary metabolomic profile in mice after exposure to four types of radiation. (**A**) singular value decomposition-based principle component analysis (PCA) constructed in R showing the distinct separation of the metabolomic profiles of control mice (**red** circles) from that of mice exposed to ionizing radiation (IR) using the ions present in at least 70% of the samples. This PCA also highlights the similarities in the metabolomic signatures of the 4 exposure types; X-ray irradiation at 1.1 Gy/min (HDR) (**dark blue** circles), X-ray irradiation at 3.0 mGy/min (LDR) (**yellow** circles), internal ^137^Cs (**purple** circles), and internal ^90^Sr (**light blue** circles). While the metabolomic signatures of ^90^Sr and ^137^Cs exposures match each other more closely, they do overlap with HDR and LDR X-ray irradiations; (**B**) Venn diagram showing the distribution of statistically significant metabolic pathways among the different exposure types. This diagram shows the number of Kyoto Encyclopedia of Genes and Genomes (KEGG) pathways with statistically significant perturbations after the above four IR exposures. The blue box highlights the common KEGG pathways, which had statistically significant changes in the urinary excretion of at least one of their intermediates in all four exposure scenarios. This figure shows overall metabolic perturbations (increase or decrease) in these pathways induced by all exposure types.

The similarities and differences in the perturbations induced by the following IR sources in the urinary metabolomic profiles of mice were initially evaluated: internal ^137^Cs, internal ^90^Sr, X-ray irradiation at 1.1 Gy/min, X-ray irradiation at 3.0 mGy/min. The principle component analysis (PCA) plot in Figure 1A shows that, while exposure to all four types of IR cases above induced statistically significant and distinct perturbations in the overall urinary metabolomic signature in mice compared to that of the control mice (red circles), the differences in these perturbations in each exposure case is subtle as shown by the close clustering of the metabolomic profiles on the right hand side of the PCA plot. In addition, the Venn diagram in Figure 1B highlights, that although differences exist among the metabolic perturbations induced by each of the four exposure scenarios, many of the perturbed pathways annotated via Kyoto Encyclopedia of Genes and Genomes (KEGG) are shared among them (marked by a blue box and arrow pointing to the table of common KEGG pathways). However, this does not mean that these exposures induce the same changes (increase/decrease) in the same intermediates of these metabolic pathways. Rather, they can have different impact (increase/decrease/no change) on the urinary concentration of various metabolic intermediates in a pathway. Ions identified here as calcitroic acid and citrulline are two metabolite examples of how different exposure types (internal emitter *vs.* X-ray irradiation) and dose rate in the case of X-ray irradiation (LDR *vs.* HDR) modulate exposure responses.

**Figure 2:**
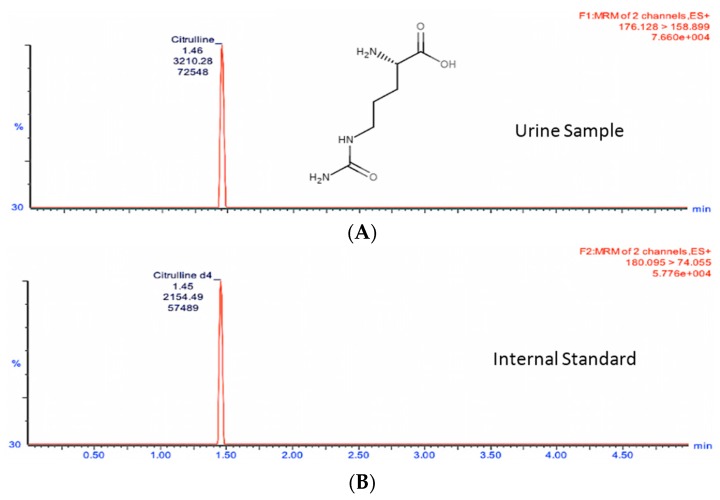
Representative UPLC-MS/MS chromatograms of endogenous urinary citrulline detected at 91.5 ng/mL and citrulline-d_4_ at 150 ng/mL (Internal Standard). The retention time is presented on the *x*-axis and the *y*-axis represent the ion count for the channel. The mass charge ratio of the parent and daughter ions, as well as the ion count for each channel is shown. The peaks represent the most abundant ions that were used for quantification. Multiple reaction monitoring (MRM) transition chromatogram of (**A**) *m*/*z* 176.1→158.9 for citrulline and (**B**) *m*/*z* 180.1→74.1 for spiked neat internal standard citrulline-d_4_ used for quantitation.

**Figure 3:**
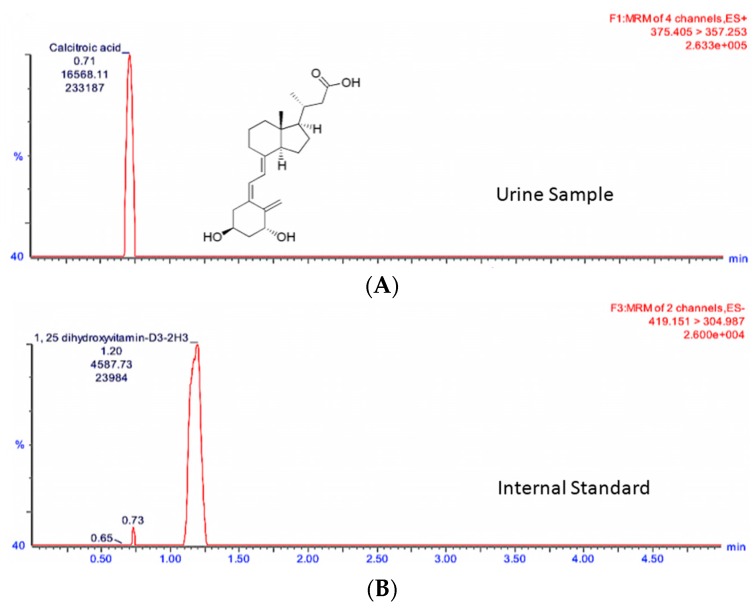
Representative UPLC-MS/MS chromatograms of calcitroic acid standard spiked in urine detected at 250 ng/mL and 1α,25-dihydroxyvitamin-D_3_-d_3_ at 500 ng/mL (Internal Standard). The retention time is presented on the *x*-axis and the *y*-axis represent the ion count for the channel. The mass charge ratio of the parent and daughter ions, as well as the ion count for each channel, is shown. The peaks represent the most abundant ions that were used for quantification. MRM transition chromatogram of (**A**) *m*/*z* 375.4→357.3 for calcitroic acid and (**B**) *m*/*z* transition 419.2→305.0 for spiked neat internal standard 1α,25-dihydroxyvitamin-D_3_-d_3_ used for quantitation. Note that quantitation only involved peak area at 1.20 min and excluded that of the peak at 0.73 min.

In order to study the changes in these two metabolites in a quantitative manner, we developed and optimized MRM methods for citrulline and calcitroic acid in mouse urine using a Waters (Milford, MA, USA) tandem quadrupole TQ-S mass spectrometer (Table 1). As shown in Figure 2 and Figure 3, the target analytes and the appropriate internal standards (IS) for citrulline and calcitroic acid provided high sensitivity and response in positive electrospray ionization (ESI) mode. Figure 2 shows the citrulline MRM transition chromatogram of the analyte (A) at *m*/*z* transition 176.1→158.9 and (B) *m*/*z* transition 180.1→74.1 for the spiked neat internal standard citrulline-d_4_ which was also used for quantitation. Similarly, Figure 3 shows the chromatogram of MRM transition at *m*/*z* 375.4→357.3 for calcitroic acid (A) and (B) *m*/*z* transition 419.2→305.0 for the spiked neat internal standard, 1α,25-dihydroxyvitamin-D_3_-d_3_. It is important to note that quantitation of calcitroic acid only involved the area of the peak at 1.20 min and excluded that of the peak at 0.73 min.

**Figure 4:**
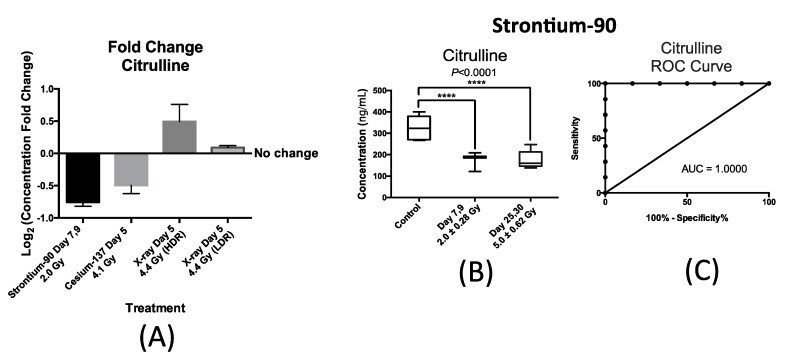
(**A**) changes in the urinary concentration of citrulline from mice after various exposure types. The *y*-axis represents log_2_ fold change after exposure and *x*-axis represents each of the three exposure senarios; ^90^Sr exposure at days 7–9 after exposure at cumulative dose of 2.0 Gy, ^137^Cs at day 5 after exposure at cumulative dose of 4.1 Gy, and X-ray at day 5 post 4.4 Gy exposure at high dose rate (HDR) of 1.1 Gy/min and low dose rate (LDR) of 0.00309 Gy/min. ^90^Sr and to a lesser extent ^137^Cs exposure induced a decrease in the urinary levels of citrulline, while X-ray exposure (HDR) had an opposite effect. No statistically significant change in the urinary concentration of calcitroic acid was detected after LDR exposure; (**B**) box plot representation of urinary concentration of citrulline after ^90^Sr-exposure. The calculated mean ± standard error of the mean (SEM) for the control mice was 326.3 ± 24.6 ng/mL, for days 7–9 at cumulative skeleton dose of 2.0 Gy was 182.8 ± 10.7 ng/mL, and for days 25–30 at 5.0 Gy was 176.1 ± 18.8 ng/mL. Each study group contained at least six samples. Concentration fold change was calculated by the log_2_ ratio of urinary concentration of citrulline in mice exposed to ^90^Sr to that of citrulline in control mice at each time-point; (**C**) ROC analysis of citrulline at time-point days 7–9 after ^90^Sr-exposure at cumulative average dose of 2.0 Gy compared to controls shows the robustness of the biomarker classification. The predictive power of the biomarker at cutoff of 268.7 ng/mL is at 83.3% specificity and 100% sensitivity. (****) denotes the statistical significance in terms of *p*-value (*p*-value < 0.0001) of the change in concentration of citrulline after exposure to ^90^Sr in mice at each of the two time-points.

Once liquid chromatography mass spectrometry (LCMS) method and MRM transitions (fragment structures for citrulline and calcitroic acid transitions provided in Supplementary Materials Figure S1) were optimized for each analyte, the internal standards were used to calculate urinary concentration of the analytes for each exposure scenario at the designated time/dose points. Figure 4 summarizes our findings with respect to urinary excretion of citrulline in response to internal exposure to ^90^Sr and ^137^Cs and X-ray exposure at the dose rate of 1.1 Gy/min (HDR) at similar time-points; within one week after exposure. This time-point was chosen to accommodate a real-world nuclear/radiological disaster where exposure monitoring for many will occur in the first week. Thus, the candidate IR markers of exposure need to have a stable and persistent signal during this time to help medical responders assess exposure. The X-ray exposure at the dose rate of 3.0 mGy/min (LDR) induced no statistically significant changes in the urinary concentration of citrulline post exposure to 4.4 Gy of X-ray exposure delivered at 3.0 mGy/min over a 24 h period as shown in Figure 4A. This plot also shows the statistically significant changes in the urinary concentration of citrulline in mice after exposure to ^90^Sr (internal exposure) after seven days at cumulative dose of 2.0 Gy, ^137^Cs (internal exposure) at day 5 after exposure at cumulative dose of 4.1 Gy, and X-ray at day 5 post 4.4 Gy exposure at high dose rate (HDR) of 1.1 Gy/min. The *y*-axis, log_2_ fold change, signifies the magnitude of change in the urinary excretion of citrulline after exposure and was calculated by the log_2_ ratio of urinary concentration of citrulline in the exposed mice exposed to that of citrulline in control mice at each time-point. The *x*-axis represents each of the four exposure scenarios. Exposure to ^90^Sr and to a lesser extent to ^137^Cs induced a decrease in the urinary levels of citrulline, while X-ray exposure delivered at HDR had the opposite effect. The decrease in the urinary levels of citrulline is thus specific to the two internal emitters with ^90^Sr having a more persistent effect (Figure 4B) than ^137^Cs (Supplementary Materials Figure S2). Thus, we further measured and tested the feasibility and predictability of citrulline in the case of ^90^Sr exposure. Figure 4B shows a box plot representation of the significant and persistent drop in the urinary concentration (ng/mL) of citrulline after ^90^Sr-exposure throughout the 30-day study. The calculated mean concentration ± SEM for citrulline in the control mice was 326.3 ± 24.6 ng/mL, while this number of the exposed mice at days 7–9 at the cumulative skeleton dose of 2.0 Gy was 182.8 ± 10.7 ng/mL, and 176.1 ± 18.8 ng/mL for days 25–30 at 5.0 Gy. These calculations were based on measurements in at least six mice per study group. The persistent decrease in the urinary concentration of citrulline is clearly evident in this panel, dropping to almost one half of the control levels at days 25–30 after ^90^Sr-exposure. In the case of ^137^Cs exposure, the urinary excretion of citrulline drops by almost 30% at five days after exposure at cumulative dose of 4.1 Gy (Supplementary Materials Figure S2). This is the largest and most statistically significant drop in citrulline’s urinary levels at this time/dose point, and its levels climb back to its pre-exposure levels by the end of the 30-day study. In the case of HDR exposure, statistical significance was reached at 4.4 Gy five days after exposure with after exposure excretion levels of citrulline at 1.4 times its pre-exposure levels. Figure 4C displays a Receiver Operating Characteristic (ROC) curve for citrulline at time-point day 7 after exposure at cumulative average dose of 2.0 Gy compared to controls showing the predictive power of this metabolite in exposure classification. In this plot, the area under the curve (AUC) demonstrates the sensitivity and specificity of the biomarker from 50% chance up to 100%. The exposure assessment ability of citrulline at the cutoff value of 268.7 ng/mL (days 7–9) and *p*-value of 0.0062 had a specificity of 83.3% and sensitivity of 100%.

**Figure 5:**
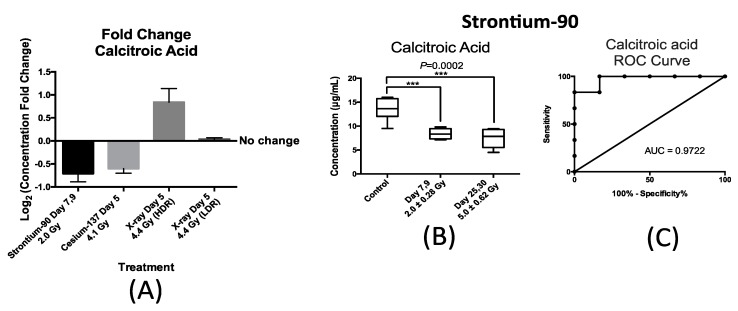
(**A**) changes in the urinary concentration of calcitroic acid from mice after various exposure types. The *y*-axis represents log_2_ fold change after exposure and *x*-axis represents each of the three exposure senarios; ^90^Sr exposure at days 7–9 after exposure at cumulative dose of 2.0 Gy, ^137^Cs at day 5 after exposure at cumulative dose of 4.1 Gy, and X-ray at day 5 post 4.4 Gy exposure at high dose rate (HDR) of 1.1 Gy/min min and low dose rate (LDR) of 0.00309 Gy/min. ^90^Sr and to a lesser extent ^137^Cs exposure induced a decrease in the urinary levels of calcitroic acid, while X-ray exposure (HDR) had an opposite effect. No statistically significant change in the urinary concentration of calcitroic acid was detected after LDR exposure; (**B**) box plot representation of urinary concentration of calcitroic acid from mice after ^90^Sr-exposure. The calculated mean ± SEM for the control mice was 13.6 ± 0.96 μg/mL, for days 7–9 at cumulative skeleton dose of 2.0 Gy was 8.4 ± 0.46 μg/mL, and for days 25–30 at 5.0 Gy was 7.5 ± 0.90 μg/mL. Each study group contained at least 6 samples; (**B**) concentration fold change was calculated by the log_2_ ratio of urinary concentration of calcitroic acid in mice exposed to ^90^Sr to that of calcitroic acid in control mice at each time-point. This figure clearly shows a persistent and statistically significant decrease in the urinary concentration of calcitroic acid after ^90^Sr-exposure; (**C**) ROC analysis of calcitroic at time-point days 7–9 after exposure at cumulative average dose of 2.0 Gy compared to controls shows that the sensitivity and specificity at cutoff of 11.35 μg/mL are 100% and 83.3% respectively. (***) denotes the statistical significance in terms of *p*-value (*p*-value = 0.0002) of the change in concentration of calcitroic acid after exposure at each of the two time-points.

Similarly, the urinary excretion of calcitroic acid was quantified post exposure to both internal emitters and to LDR and HDR X-ray irradiation. In the case of urinary excretion of calcitroic acid, exposure to X-ray exposure at the dose rate of 3.0 mGy/min (LDR) also did not result in a statistically significant change (Figure 5A). Panel A in Figure 5 also shows the changes in the urinary concentration of calcitroic acid from mice after exposure to ^90^Sr at day 7 after exposure at cumulative dose of 2.0 Gy, ^137^Cs at day 5 after exposure at cumulative dose of 4.1 Gy, and X-ray at day 5 post 4.4 Gy exposure at high dose rate (HDR) of 1.1 Gy/min. This plot was constructed similarly to Figure 4A as described above. This plot shows that exposure to ^90^Sr and to a lesser extent ^137^Cs exposure induced a decrease in the urinary levels of calcitroic acid, while X-ray exposure (HDR) had an opposite effect. As with citrulline, the decrease in the urinary excretion of calcitroic acid after ^90^Sr-exposure was persistent throughout the 30-day study (Figure 5B). The box plot in Figure 5B represents the urinary concentration of calcitroic acid in control and exposred mice. In the control mice, the urinary concentration of calcitroic acid was 13.6 ± 0.96 μg/mL, for days 7–9 at cumulative skeleton dose of 2.0 Gy it was 8.4 ± 0.46 μg/mL, and for days 25–30 at 5.0 Gy, it was 7.5 ± 0.90 μg/mL. However, in the case of ^137^Cs exposure, the decrease in urinary excretion levels of calcitroic acid showed a dose dependent response (Supplementary Materials Figure S2), unlike with citrulline. Both internal emitters induced a persistent decrease in the urinary concentration of calcitroic acid with a dose/time dependency in the case of ^137^Cs exposure. On the other hand, urinary excretion levels of calcitroic acid increased by 15% at five days after HDR-exposure (4.4 Gy). Exposure to ^90^Sr induced the largest change in the levels of calcitroic acid thus we chose to further analyze these samples to assess the predictive power of calcitroic acid as an IR exposure marker. Figure 5C depicts an ROC analysis of calcitroic at time-point days 7–9 after exposure at cumulative average dose of 2.0 Gy compared to controls. Calcitroic acid had an AUC of 0.97 at this time/dose point at the cutoff value of 11.4 μg/mL and *p*-value of 0.0065 at 83.3% specificity and 100% specificity.

## 3. Discussion

This study focuses on the identification and quantitative assessment of two urinary biomarkers of IR exposure and complements our previous investigations into the metabolomic profile of urine from mice internally exposed to ^90^Sr and ^137^Cs and to X-ray beam radiation. Exposure to internal emitters such as ^90^Sr and ^137^Cs is a well-recognized risk after a nuclear/radiological disaster. While more attention has been given to metabolomic perturbations post exposure to X-ray, particularly at 1 Gy/min, less is known about how dose rate modulates these perturbations and the effects of internal exposure to internal emitters on metabolism. Our initial comprehensive analyses of the changes in the urinary metabolomic profiles in mice after exposure to X-ray; radiation at the LDR and the HDR as well as to ^90^Sr and ^137^Cs demonstrated that these exposures can induce similar perturbations in the general metabolomic profile of urine in mice as seen in Figure 1A. While these four different types of exposures induce changes in many of the same metabolic pathways (Figure 1B), such as pathways associated with energy metabolism, they have varying effects (decrease/increase) on different intermediates in these pathways. It is important to note that the changes observed were the direct result of exposure to various IR sources. We did not detect any adverse events or observations for in the food/water consumption and the weight of the mice thoughout the 30-day studies. While several IR-sensitive metabolites have already been chemically characterized through conventional tandem MS experiments in our previous work [7], two robust features with highly statistically significant urinary changes could not be chemically identified and further characterized due to lack of commercial chemical standards and unavailability of quantitative approaches in urine until now. Here, we describe the identification and the quantitative methods used for the characterization of these two promising urinary IR-markers, calcitroic acid and citrulline, particularly in the case of internal emitters. Optimized MRM methods were then used for calculating the changes in their concentration in mouse urine after each exposure case (Figure 4 and Figure 5 and Supplementary Materials) as summarized in Table 2.

**Figure 6:**
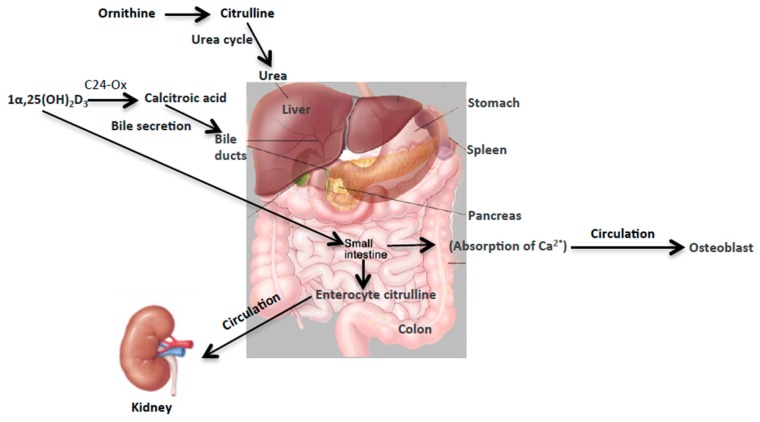
Overall metabolism of citrulline in the urea cycle and of calcitroic acid secretion into the bile. Citrulline is an important intermediate of the urea cycle in the liver and is primarily synthesized from arginine and glutamine in the enterocytes. Citrulline is essential for the synthesis of arginine, which in turn produces nitric oxide. On the other hand, calcitroic acid has a less defined biological role. Calcitroic acid is an end product of 1α,25-(OH)_2_D_3_ metabolism through a C-24 oxidation by mitochondrial CYP24 enzyme and is secreted into the bile [11]. Osteoblasts, kidneys and intestine metabolize 1α,25-(OH)_2_D_3_ through this oxidation pathway, which is an important regulatory pathway for dihydroxyvitamin-D_3_.

The first metabolite to be studied was citrulline, which is an alpha-amino acid and is mainly synthesized by small bowel enterocytes. We observed a significant decrease in the urinary concentration of citrulline after internal exposure to ^90^Sr and ^137^Cs in mice (Figure 4 and Figure S1). While we did not detect any statistically significant changes in the urinary excretion of citrulline at 4.4 Gy X-ray irradiation delivered at low dose rate (LDR) of 3.0 mGy/min, we did observe a statistically significant increase post 4.4 Gy X-ray irradiation at high dose rate (HDR) of 1.1 Gy/min (Figure 4). Citrulline is essential for the synthesis of arginine (Figure 6), which in turn produces nitric oxide. Citrulline is released by the small intestine continuously, thus perturbations in its concentration may be a good marker of intestinal dysfunction [27]. A decrease in citrulline levels is clinically relevant as it reflects a decrease in the functional mass of enterocytes [28,29] and radiation-induced intestinal epithelial damage [30,31]. In fact, citrullinaemia is a more sensitive and specific test than the sugar permeability test for assessing small bowel toxicity and enterocyte function [32]. Thus, the total concentration of citrulline at any given time may be used in a predictive manner for gut injury. This could potentially be indicative of an early but transient damage to the small bowel in the case of ^137^Cs as the decreased citrulline levels return to baseline 30 days after exposure (Figure S2), while this damage may be more persistent in the case of ^90^Sr (Figure 4B). In the case of LDR X-ray irradiation, the repair and adaptation mechanism over time (3 mGy/min) may prevent damage to the gut epithelium, which may be why we did not detect statistically significant changes in urinary citrulline concentration after LDR exposure unlike with HDR exposure. Although the metabolomic signatures of LDR and HDR share many similarities with each other and those of internal emitters, there are subtle yet important differences in the metabolic perturbations each exposure type induces. The case of citrulline is an example of such a case in the urinary excretion profile of metabolites due to exposure route and dose rate. Our previous gene expression study of blood white cells of the mice exposed to internal emitters showed a slight yet statistically significant increase in the expression of *NOS1* after ^90^Sr-exposure at days 25–30 while no changes were found with respect to ^137^Cs or X-ray irradiation. NOS1 is a calcium regulated isomer of NOS and responsible for the production of citrulline. While this observation is intriguing, it is very important to keep in mind the complexity of citrulline metabolism. Thus, much work is needed to draw any conclusion here between the decrease in the concentration of citrulline after exposure and the observed overexpression levels of *NOS1*.

Lastly, we analyzed the urinary concentration of calcitroic acid, which remained steady with no statistically significant changes after LDR exposure, yet showed distinct increase after exposure to HDR γ irradiation compared to a significant decrease after exposure to the two internal emitters (Figure 5A). Calcitroic acid is the terminal product of the deactivation of 1a,25-dihydroxyvitamin D_2_. Vitamin D_2_ and D_3_ are metabolized in a similar fashion producing hormonally active form of the 1α,25-(OH)_2_D_2_. The synthesis of calcitroic acid from 1α,25-(OH)_2_D_2_ is carried out by the isomers of the cytochrome P450 (CYP) in a multi-step catalytic process. CYP4 proteins metabolize vitamin D and are also important in chemical and environmental defense. A gene expression study carried out on the blood of mice exposed to ^90^Sr showed a lower expression of two *CYP* genes, *Cyp4f18* and *Cyp4v3* while an increase was observed in the expression of *Cyp4v3* post HDR X-ray irradiation [15,16,17]. Although it is hard to draw direct conclusions from the gene expression and the metabolomics data, significant decrease in the urinary concentration of calcitroic acid in the case of ^90^Sr-exposure, and the increase in the case of HDR X-ray irradiation, may be indicative of abnormalities in the regulatory oxidation pathway of vitamin D_3_ and linked to the changes in the expression of *Cyp4v3* [18]. In the case of ^137^Cs changes in the expression of Cytochrome *P450* gene, *Cyp2r1*, was induced when rats were exposed to ^137^Cs in their drinking water for up to three months [33]. This is further evidence that chronic exposure to ^137^Cs affects vitamin D metabolism by inducing changes in the CYP enzymes. Collectively, these studies show that vitamin D synthesis and catabolism are radiosensitive metabolic pathways. Further proof can be found in bone mineral density data from the workers of the Chernobyl accident [34] and bone cancer data reported in previous studies on ^137^Cs-treated animals [35]. The involvement of vitamin D metabolism in radiation injury has been documented by several gene expression studies as noted above; however, limited information is available on the mechanism through which ionizing radiation modules vitamin D synthesis and catabolism. While previous untargeted and targeted metabolomic studies by us and other groups have shown severe changes in the energy and lipid metabolism after radiation exposure [1,2,3,4,5,6,7,8,9], more work is needed to explore the effects of exposure on vitamin D metabolism. While the metabolomic data presented here and the results of the previous gene expression studies independently suggest changes in the vitamin D metabolism, the link between these two platforms may be achieved by future proteomic approaches on CYP and other enzymes associated with vitamin D metabolism.

**Figure 7:**
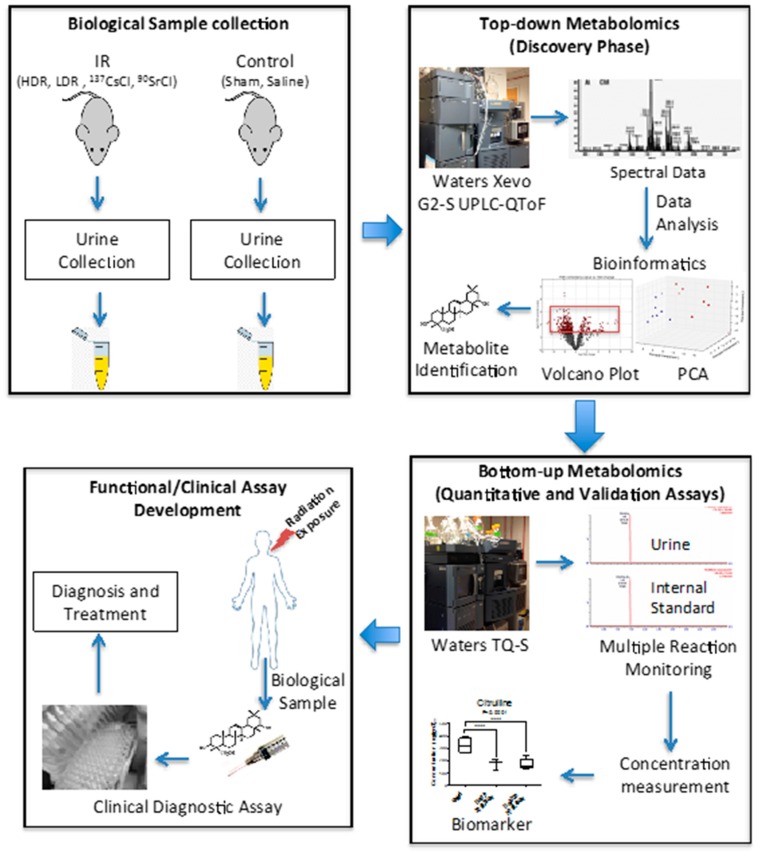
Overview of the untargeted and the quantitative studies. Urine samples were collected at indicated time points from mice exposed to various IR sources; internal ^90^Sr, internal ^137^Cs, X-ray HDR, and X-ray LDR The samples were initially subjected to untargeted metabolomic profiling using Waters Xevo G2-S UPLC-QToF (Waters, Milford, MA, USA). The acquired data were deconvoluted and analyzed using various bioinformatics tools. A panel of statistically significant metabolite markers were selected based on their clear IR responses, robustness of responses, magnitude of responses, and biological importance. Citrulline and calcitroif acid matched these criteria and thus were selected for quantitative assessment using Waters tandem quadrupole TQ-S. Multiple reaction monitoring methods were developed with the help of appropriate internal standards. The results of such quantitative study are promising as they have the potential to be part of functional assays with clinical applications to assist medical workers assess exposure in a population after a radiological and/or nuclear event.

This study highlights the unique changes in the concentration of two urinary metabolites, citrullin and calcitroic acid, post exposure to internal emitters ^90^Sr and ^137^Cs, and X-ray irradiation (HDR and LDR). These robust changes, particularly in the case of internal emitters hint at perturbations in the overall metabolism of citrulline in the urea cycle and of calcitroic acid secretion into the bile (Figure 6). Though the two biomarkers reported here are involved in two distinct metabolic processes together they may serve as reliable yet independent predictors of exposure, particularly for exposure to internal emitters. The observed differences in the exposure responses with respect to each of the two markers as noted in Table 2 are due to differences in radiation quality, tissue exposure patterns, and varying dose rate, particularly in the case of the internal emitters. Exposure at low dose rates is suggested to dampen the metabolomic responses as it allows for ongoing DNA repair and is perhaps the reason why no changes were detected in the case of LDR exposure. Although exposure to ionizing radiaon induces changes in similar pathways with respect to internal and external exposure sources, the results of our previous studies on HDR exposure [5] and exposure to ^137^Cs [6] and ^90^Sr [7] also suggested that the urinary metabolomic responses in mice induced by exposure to the two internal emitters have more in common with each other than with HDR exposure. This may be due to varying low dose rates and tissue exposure patterns in the case of the two internal emitters. This study was built on our previous comprehensive qualitative assessment of urinary metabolomic perturbations in mice after exposure to various types of IR exposures (top two panels of Figure 7). These qualitative and discovery-phase studies highlighted the robust and statistically significant changes in the urinary excretion of two ions and laid the foundation for their identification and quantitative assessment as reported in the current study (bottom panels of Figure 7). This study also aimed at the refinement of the previously established exposure metabolomic profiles and has the promise of clinical and biodosimetry applications. A refined and validated exposure signature in easily accessible biofluids will help clinical laboratories assess individual exposure after a nuclear/radiological event. Quick yet accurate high-throughput biodosimetry depends on exposure and dose specific biomarkers. Thus, candidate biomarkers such as those reported here are promising and, once independently validated, may be used as high throughput biodosimetry countermeasures.

## 4. Materials and Methods

UPLC-grade solvents were purchased from Fisher Scientific (Hanover Park, IL, USA). Citrulline was obtained from Sigma-Aldridge (St. Louis, MO, USA) and calcitroic acid was ordered from Toronto Research Chemicals Incorporated (Toronto, ON, Canada). Deuterium labeled citrulline (d_4_) was obtained from Cambridge Isotope Laboratories (Tewksbury, MA, USA) and 1α,25-Dihydroxyvitamin D_3_ (d_3_) was purchased from Isosciences (King of Prussia, PA, USA).

### 4.1. Animal Experiment and Sample Collection

We have previously described the details of the animal study and urine collection procedure [5,6,7]. Briefly, in the case of the internal emitters, C57Bl/6 (Charles River Laboratories, Frederick, MD, USA) mice at approximate age of 10–12 weeks old, under applicable federal and state guidelines and with approval by the Lovelace Respiratory Research Institute (LRRI) (Albuquerque, NM, USA) Institutional Animal Care and Use Committee (IACUC), were quarantined for 14 days upon receipt and prior to exposure to ^90^Sr or ^137^Cs. The radionulclides were administered intravenously by tail vein injection. Mice were then housed individually in microisolator cages with lead shielding. After treatment, all animals had unlimited access to Teklad Certified Global Rodent Diet 2016 (Harlan^®^ Laboratories Inc., Madison, WI, USA) and water. No adverse effects were noted for any of the animals during the course of the study. On the scheduled necropsy days, animals were euthanatized and urine was collected directly from the bladder with a needle and syringe. In the case of X-ray irradiation, under appropriate IACUC approval by Columbia University Medical Center (New York, NY, USA), male C57BL/6N mice, aged 8–10 weeks were exposed to HDR IR by being placed in a box containing bedding and irradiated in an X-ray machine (X-Rad 320, Precision X-ray Inc, Branford, CT, USA) with a dose rate of 1.03 Gy/min. For total body LDR irradiations, the mice were placed in a specially designed mouse box with prior 24 h acclimation, and placed in the X-ray machine at 22 °C (±0.5 °C) with humidity at 30%–40%. LDR irradiation was at a dose rate of 0.00309 Gy/min. The LDR mice were exposed over a pre-determined time period at 0.00309 Gy/min to achieve the desired dose (e.g., 24 h for the dose of 4.4 Gy). We created a custom Thoraeus filter (1.25 mm Sn, 0.25 mm Cu, 1.5 mm Al) that was used to perform HDR and LDR experiments using the same quality of X-rays. This filter provides a dose rate of 1.03 Gy/min at 40 cm SSD (source to surface distance) and 3.09 mGy/min at the maximum SSD. Urine collections was performed in metabolic cages (Techniplast USA, Exon, PA, USA) by placing each mouse in a single metabolic cage and collecting over a period of 24 h at day 2 and day 5 after irradiation.

### 4.2. Sample Preparation and Mass Spectrometry Analysis

Urine samples, 20 μL in volume, were prepared by dilution 1:2 in 50% acetonitrile containing internal standards as described below, followed by centrifugation at maximum speed at 4 °C and recovery of the supernatant. The stock solutions for citrulline, internal standard citrulline-d4 (approximately 1 mg/mL each) and calcitroic acid and internal standard 1α,25-dihydroxyvitamin-D_3_-d_3_ were prepared in methanol and water, respectively. The serial dilutions for each of the standards were done for the study separately in methanol/LC grade water (50:50). Preparation of the calibration curve standards and quality samples (QC) were done by mixing the stock solutions in blank matrix. The final calibration concentration for citrulline ranges from 1 to 1000 ng/mL and for calcitroic acid ranges from 100 to 2000 ng/mL The high, medium and low QC concentration for citrulline are 750, 75 and 7.5 ng/mL respectively. The high, medium and low QC concentration for calcitroic acid are 1800, 1200 and 120 ng/mL, respectively. The targeted quantitation of citrulline and calcitroic acid was performed using multiple reactions monitoring mass spectrometry in urine. The samples (injection volume of 5 μL) were resolved on an Acquity UPLC BEH HILIC 1.7 µm, 2.1 × 100 mm column online with a triple quadrupole mass spectrometer (Xevo-TQ-S, Waters Corporation, Milford, MA, USA) operating in the multiple reaction monitoring (MRM) mode (LC chromatograms, Column conditions and mobile phase gradients are specified in Table 1 and Tables S1–S4). The sample cone voltage and collision energies were optimized for both analytes to obtain maximum ion intensity for parent and daughter ions using “IntelliStart” feature of MassLynx software (Waters Corporation, Milford, MA, USA). The instrument parameters were optimized to gain maximum specificity and sensitivity of ionization for the parent (*m*/*z* = 176.12 (Citrulline)) and daughter ions (*m*/*z* = 158.89 (Citrulline)). Signal intensities from all MRM Q1/Q3 ion pairs for both analytes were ranked to ensure selection of the most intense precursor and fragment ion pair for MRM-based quantitation. This approach resulted in selection of cone voltages and collision energies that maximized the generation of each fragment ion species; the MRM parameters are specified in Table 1 and Tables S1–S4. The metabolite ratios were calculated by normalizing the peak area of endogenous metabolites within samples normalized to the internal standard. Analysis was performed with a six- to eight-point calibration curve, the sample queue was randomized and solvent blanks were injected to assess sample carryover (Figures S3 and S4). The analyte concentration linear range, recovery, accuracy, precision, and inter-batch repeatibility were also calculated (Tables S5–S11 and Figures S5 and S6). The matrix effect for each analyte and internal standard was determined by comparing the peak area in the presence of matrix measure by analyzing the blank matrix spiked after extraction with analyte to the peak area in the absence of the matrix (pure solution of the analyte). No significant change in the area was observed. The MRM data were processed using TargetLynx 4.1 (Waters, Milford, MA, USA). The relative quantification values of analytes were determined by calculating the ratio of peak areas of transitions of samples normalized to the peak area of the internal standard. These values for citrulline in the case of ^90^Sr exposure were calculated (mean ± SEM) for the control mice at 326.3 ± 24.6 ng/mL, for days 7–9 at cumulative skeleton dose of 2.0 Gy at 182.8 ± 10.7 ng/mL, and for days 25–30 at 5.0 Gy at 176.1 ± 18.8 ng/mL. The values in the case of calcitroic acid for the control mice was 13.6 ± 0.96 μg/mL, for days 7–9 at cumulative skeleton dose of 2.0 Gy was 8.4 ± 0.46 μg/mL, and for days 25–30 at 5.0 Gy was 7.5 ± 0.90 μg/mL.

### 4.3. Statistical Analysis

Our initial statistical analysis was carried out in MetaboLyzer (Washington, DC, USA) as described previously [36]. Briefly, MetaboLyzer was used to select ions (*m*/*z*) that were present in at least 70% of the samples in all study groups with nonzero abundance values (complete-presence ions). This data was used to construct the singular value decomposition-based PCA in Figure 1A using standard R graphics package. The data were then separately log transformed and analyzed using the nonparametric Mann–Whitney U test for statistical significance (*p* < 0.05). The log transformed statistically significant complete-presence ion data were further utilized for principal component analysis (PCA) via singular value decomposition for the purpose of data visualization. Statistical significance testing for ions with nonzero abundance values in at least 70% of the samples in only one group (referred to as partial-presence ions) were analyzed as categorical variables for presence status (*i.e.*, nonzero abundance) via Fisher’s exact test (*p* < 0.05). KEGG pathway annotatations for the statistically significant complete and partial presence ions were used to construct the Venn diagram in Figure 1B.

Because both *m*/*z* at 176.1 (citrulline) and at 375.4 (calcitroic acid) provided the strongest and most persistent signal throughout the ^90^Sr study, we used these data to test the feasibility and predictability of these markers once they were chemically identified and quantified. The raw data from 7 mice (time-point days 7–9) for the statistically significant ions, citrulline and calcitroic acid, were utilized for constructing a receiver operating characteristic (ROC) curve in GraphPad Prism 6 statistical software (GraphPad Software INC, La Jolla, CA, USA). The sensitivity and specificity of these two markers for ^90^Sr exposure assessment were calculated at the cutoff value. In the ROC curves, the area under the curve (AUC) is representative of the ability of the marker to discriminate between control and exposed groups. The *p*-values were two-tailed and at less than 0.05 were considered statistically significant.

## 5. Conclusions

Here, we identified and quantitated two urinary biomarkers, citrulline and calcitroic acid, for IR exposure in four exposure scenarios; internal exposure to ^90^Sr and ^137^Cs, and external γ irradiation at HDR and LDR. Calcitroic acid is a vitamin D metabolite while citrulline is a produced by arginine in the enterocytes, thus its levels signal gut dysbiosis. Upon further and independent validation, these two urinary metabolites have the potential to be used in exposure assessment after a nuclear/radiological disaster. This study is an example of the quantitative power of mass spectrometry-based metabolomics as a high-throughput platform via which clinical exposure assays may be developed and implemented in the case of such disasters. In addition, this quantitative study provided further proof that, although different types of IR exposure induce similar overall metabolomic perturbations in the urine of mice, there are subtle differences based on the route of exposure (internal/external) and dose rate (LDR/HDR).

## Figures and Tables

**Table 1:** Experimental parameters for quantitation of citrulline and calcitroic acid in urine. For each analyte, the first transition was used as quantitative transition and the second was used as confirmatory transition.

Metabolites	Parent Ion	Fragment Ion	Cone Voltage (V)	Collision Energy (V)
Citrulline	176.12	158.89	32	20
	176.12	69.82	32	8
Citrulline d_4_	180.09	117.20	16	24
	180.09	74.05	16	14
Calcitroic acid	375.34	339.33	30	38
	375.34	121.09	30	12
1α,25-Dihydroxyvitamin D_3_-d_3_	419.15	175.02	54	28
	419.15	304.98	54	14

**Table 2:** Summary of studies on radiation-induced changes in the concentration of citrulline and calcitroic acid in urine. “No change” means no statistically significant changes in the urinary excretion of metabolites were detected post-exposure.

Metabolite	Matrix	Change in Concentration	Type of Exposure	Organism	Dose, Time Post-Exposure
Citrulline	Urine	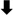	^90^Sr, internal	Mouse	2.0 Gy, Days 7,9
	Urine	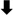	^137^Cs, internal	Mouse	4.1 Gy, Days 5
	Urine	No change	LDR (0.00309 Gy/min), X-ray	Mouse	4.4 Gy, Days 5
	Urine	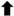	HDR (1.1 Gy/min), X-ray	Mouse	4.4 Gy, Days 5
Calcitroic acid	Urine	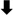	^90^Sr, internal	Mouse	2.0 Gy, Days 7,9
	Urine	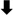	^137^Cs, internal	Mouse	4.1 Gy, Days 5
	Urine	No change	LDR (0.00309 Gy/min), X-ray	Mouse	4.4 Gy, Days 5
	Urine	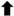	HDR (1.1 Gy/min), X-ray	Mouse	4.4 Gy, Days 5

^90^Sr, Strontium-90; ^137^Cs, Cesium-137; LDR, low dose rate; HDR, high dose rate.

## Supplementary Materials

Supplementary materials can be found at http://www.mdpi.com/1422-0067/17/5/782/s1.

## Abbreviations

UPLC/MS/MS	Ultra-performance liquid chromatography tandem mass spectrometry
LDR	Low dose rate
HDR	High dose rate
MRM	Multiple reaction monitoring
^90^Sr	Strontium-90
^137^Cs	Cesium-137

## References

[1] Lanz C., Patterson A.D., Slavík J., Krausz K.W., Ledermann M., Gonzalez F.J., Idle J.R. (2009). Radiation metabolomics. 3. Biomarker discovery in the urine of gamma-irradiated rats using a simplified metabolomics protocol of gas chromatography-mass spectrometry combined with random forests machine learning algorithm. Radiat. Res..

[2] Mak T.D., Tyburski J.B., Krausz K.W., Kalinich J.F., Gonzalez F.J., Fornace A.J. (2015). Exposure to ionizing radiation reveals global dose- and time-dependent changes in the urinary metabolome of rats. Metabolomics.

[3] Tyburski J.B., Patterson A.D., Krausz K.W., Slavik J., Fornace A.J., Gonzalez F.J., Idle J.R. (2009). Radiation metabolomics. 2. Dose- and time-dependent urinary excretion of deaminated purines and pyrimidines after sublethal γ-radiation exposure in mice. Radiat. Res..

[4] Johnson C.H., Patterson A.D., Krausz K.W., Kalinich J.F., Tyburski J.B., Kang D.W., Luecke H., Gonzalez F.J., Blakely W.F., Idle J.R. (2012). Radiation metabolomics. 5. Identification of urinary biomarkers of ionizing radiation exposure in nonhuman primates by mass spectrometry-based metabolomics. Radiat. Res..

[5] Goudarzi M., Mak T.D., Chen C., Smilenov L.B., Brenner D.J., Fornace A.J. (2014). The effect of low dose rate on metabolomic response to radiation in mice. Radiat. Environ. Biophys..

[6] Goudarzi M., Weber W.M., Mak T.D., Chung J., Doyle-Eisele M., Melo D.R., Brenner D.J., Guilmette R.A., Fornace A.J. (2014). Development of urinary biomarkers for internal exposure by cesium-137 using a metabolomics approach in mice. Radiat. Res..

[7] Goudarzi M., Weber W.M., Mak T.D., Chung J., Doyle-Eisele M., Melo D.R., Strawn S.J., Brenner D.J., Guilmette R.A., Fornace A.J. (2015). A Comprehensive Metabolomic Investigation in Urine of Mice Exposed to Strontium-90. Radiat. Res..

[8] Goudarzi M., Weber W.M., Mak T.D., Chung J., Doyle-Eisele M., Melo D.R., Brenner D.J., Guilmette R.A., Fornace A.J. (2015). Metabolomic and lipidomic analysis of serum from mice exposed to an internal emitter, cesium-137, using a shotgun LC-MS(E) approach. J. Proteome Res..

[9] Goudarzi M., Weber W.M., Chung J., Doyle-Eisele M., Melo D.R., Mak T.D., Strawn S.J., Brenner D.J., Guilmette R., Fornace A.J. (2015). Serum Dyslipidemia Is Induced by Internal Exposure to Strontium-90 in Mice, Lipidomic Profiling Using a Data-Independent Liquid Chromatography-Mass Spectrometry Approach. J. Proteome Res..

[10] Zimmerman D.R., Reinhardt T.A., Kremer R., Beitz D.C., Reddy G.S., Horst R.L. (2001). Calcitroic acid is a major catabolic metabolite in the metabolism of 1 α-dihydroxyvitamin D_2_. Arch. Biochem. Biophys..

[11] Inouye K., Sakaki T. (2001). Enzymatic studies on the key enzymes of vitamin D metabolism; 1 α-hydroxylase (CYP27B1) and 24-hydroxylase (CYP24). Biotechnol. Annu. Rev..

[12] Jones G. (2012). Metabolism and biomarkers of vitamin D. Scand. J. Clin. Lab. Investig. Suppl..

[13] Prosser D.E., Jones G. (2004). Enzymes involved in the activation and inactivation of vitamin D. Trends Biochem. Sci..

[14] Langberg M., Rotem C., Fenig E., Koren R., Ravid A. (2009). Vitamin D protects keratinocytes from deleterious effects of ionizing radiation. Br. J. Dermatol..

[15] Ghandhi S.A., Weber W.M., Melo D.R., Doyle-Eisele M., Chowdhury M., Guilmette R., Amundson S.A. (2015). Effect of ^90^Sr internal emitter on gene expression in mouse blood. BMC Genomics.

[16] Paul S., Ghandhi S.A., Weber W.M., Doyle-Eisele M., Melo D.R., Guilmette R., Amundson S.A. (2014). Gene expression response of mice after a single dose of ^137^CS as an internal emitter. Radiat. Res..

[17] Paul S., Smilenov L.B., Elliston C.D., Amundson S.A. (2015). Radiation Dose-Rate Effects on Gene Expression in a Mouse Biodosimetry Model. Radiat. Res..

[18] Kirischian N.L., Wilson J.Y. (2012). Phylogenetic and functional analyses of the cytochrome P450 family 4. Mol. Phylogenet. Evol..

[19] Wu G. (1997). Synthesis of citrulline and arginine from proline in enterocytes of postnatal pigs. Am. J. Physiol..

[20] Crenn P., Hanachi M., Neveux N., Cynober L. (2011). Circulating citrulline levels: A biomarker for intestinal functionality assessment. Ann. Biol. Clin. (Paris).

[21] Grimaldi D., Guivarch E., Neveux N., Fichet J., Pène F., Marx J.S., Chiche J.D., Cynober L., Mira J.P., Cariou A. (2013). Markers of intestinal injury are associated with endotoxemia in successfully resuscitated patients. Resuscitation.

[22] Pan L., Wang X., Li W., Li N., Li J. (2010). The intestinal fatty acid binding protein diagnosing gut dysfunction in acute pancreatitis: A pilot study. Pancreas.

[23] Noordally S.O., Sohawon S., Semlali H., Michely D., Devriendt J., Gottignies P. (2012). Is there a correlation between circulating levels of citrulline and intestinal dysfunction in the critically ill. Nutr. Clin. Pract..

[24] Crenn P., Neveux N., Chevret S., Jaffray P., Cynober L., Melchior J.C., Annane D. (2014). Plasma l-citrulline concentrations and its relationship with inflammation at the onset of septic shock: A pilot study. J. Crit. Care.

[25] Berdeaux A. (1993). Nitric oxide: An ubiquitous messenger. Fundam. Clin. Pharmacol..

[26] Leach J.K., Black S.M., Schmidt-Ullrich R.K., Mikkelsen R.B. (2002). Activation of constitutive nitric-oxide synthase activity is an early signaling event induced by ionizing radiation. J. Biol. Chem..

[27] Zezulová M., Bartoušková M., Hlídková E., Adam T., Kujovská Krčmová L., Červinková B., Solichová D., Zlevorová M., Cwiertka K., Friedecký D. (2015). Citrulline as a biomarker of gastrointestinal toxicity in patients with rectal carcinoma treated with chemoradiation. Clin. Chem. Lab. Med..

[28] Woo H.K., Kim E-K., Jung Y.H., Shin S.H., Kim H.-S., Choi J.-H., Kim H.-Y. (2015). Reduced early dried blood spot citrulline levels in preterm infants with meconium obstruction of prematurity. Early Hum. Dev..

[29] Crenn P., De Truchis P., Neveux N., Galpérine T., Cynober L., Melchior J.C. (2009). Plasma citrulline is a biomarker of enterocyte mass and an indicator of parenteral nutrition in HIV-infected patients. Am. J. Clin. Nutr..

[30] Lutgens L., Lambin P. (2007). Biomarkers for radiation-induced small bowel epithelial damage: An emerging role for plasma Citrulline. World J. Gastroenterol..

[31] Lutgens L.C., Deutz N.E., Gueulette J., Cleutjens J.P., Berger M.P., Wouters B.G., von Meyenfeldt M.F., Lambin P. (2003). Citrulline: A physiologic marker enabling quantitation and monitoring of epithelial radiation-induced small bowel damage. Int. J. Radiat. Oncol. Biol. Phys..

[32] Vokurka S., Svoboda T., Rajdl D., Sedláčková T., Racek J., Koza V., Trefil L. (2013). Serum citrulline levels as a marker of enterocyte function in patients after allogeneic hematopoietic stem cells transplantation-a pilot study. Med. Sci. Monit..

[33] Tissandie E., Gueguen Y., Lobaccaro J.M.A., Aigueperse J., Gourmelon F., Paquet F., Souidi M. (2006). Chronic contamination with ^137^ Cesium affects vitamin D_3_ metabolism in rats. Toxicology.

[34] Kharchenko V.P., Rassokhin B.M., Zubovskii G.A. (2001). Value of bone densitometry in the determination of vertebral mineral density in participants of the clean-up after Chernobyl accident. Med. Truda Prom. Ekol..

[35] Fritz T.E. (1972). Cesium 137 late effects of single intravenous injections in beagles. ANL 7970, Argone Natl. Lab. Annu. Rep. USA.

[36] Mak T.D., Laiakis E.C., Goudarzi M., Fornace A.J. (2014). MetaboLyzer: A novel statistical workflow for analyzing Postprocessed LC-MS metabolomics data. Anal. Chem..

