# Supplementary material for: Quantitative Metabolomic Analysis of Urinary Citrulline and Calcitroic Acid in Mice after Exposure to Various Types of Ionizing Radiation
Source: Int J Mol Sci. 2016 May 20;17(5):782. doi: 10.3390/ijms17050782 (PMC4881599; doi:10.3390/ijms17050782)
Supplement: Supplementary file 1 [file ijms-17-00782-s001.pdf]

Supplementary Materials: Quantitative Metabolomic Analysis of Urinary Citrulline and Calcitroic Acid in Mice after Exposure to Various Types of Ionizing Radiation

Maryam Goudarzi, Siddheshwar Chauthe, Steven J. Strawn, Waylon M. Weber, David J. Brenner and Albert J. Fornace Jr.

Table S1. Experimental LC-MS conditions for citrulline and calcitroic acid.

UPLC Column	Acquity UPLC BEH HILIC. 1.7 µm, 2.1 mm × 100 mm
Injection volume (µL)	5
Column temperature (°C)	45
Autosampler injection mode	Partial loop needle overfill (PLNO)

Table S2. Tune page parameters.

Instrument	Xevo-TQS
Capillary voltage (kV)	3
Cone voltage (V)	30
Source temperature (°C)	150
Desolvation temperature (°C)	500
Cone gas flow (L/Hr)	150
Desolvation gas (L/Hr)	900
Sample temperature (°C)	4
Mode of ionization	Positive

Table S3. Mobile phase gradient for citrulline. Solvent A is acetonitrile with 0.2% formic acid and solvent B is water with 0.2% formic acid.

Step	Time (min)	Flow (mL/min)	%A	%B	Curve
1	Initial	0.4	99	1	Initial
2	1	0.4	2	98	6
3	3	0.4	2	98	6
4	3.2	0.4	99	1	6
5	5	0.4	99	1	6

Table S4. Mobile phase gradient for calcitroic acid. Solvent A is methanol with 0.1% formic acid and solvent B is water with 0.2% formic acid.

Step	Time (min)	Flow (mL/min)	%A	%B	Curve
1	Initial	0.4	80	20	Initial
2	2	0.4	50	50	6
3	2.5	0.4	50	50	6
4	2.9	0.4	80	20	6
5	5	0.4	80	20	6

Table S5. Citrulline experimental linear range.

Sample Number	Sample Text	Type	Standard Concentration (ng/mL)	Retention Time (min)	Area	Concentration (ng/mL)	% Deviation *
1	Standards_1	Standard	1	1.34	4267.336	1	4.8
2	Standards_10	Standard	10	1.34	26,768.11	9.3	−6.8
3	Standards_100	Standard	100	1.34	274,634.8	100	0
4	Standards_250	Standard	250	1.34	710,569.1	255.4	2.2
5	Standards_500	Standard	500	1.34	1,356,185	503.2	0.6
6	Standards_1000	Standard	1000	1.34	2,666,926	992.1	−0.8

* % Deviation is the difference in experimentally measured standard solution concentration and the nominal concentration expressed in percentage.

Table S6. Calcitroic acid experimental linear range.

Sample Number	Sample Text	Type	Standard Concentration (ng/mL)	Retention Time (min)	Area	Concentration (ng/mL)	% Deviation*
1	Standards_100	Standard	100	0.74	6371.396	101.5	1.5
2	Standards_250	Standard	250	0.74	16,147.66	259.5	3.8
3	Standards_500	Standard	500	0.73	29,251.68	471.1	−5.8
4	Standards_750	Standard	750	0.73	43,637.5	703.5	−6.2
5	Standards_1000	Standard	1000	0.74	66,250.73	1068.8	6.9
6	Standards_2000	Standard	2000	0.74	123,612.5	1995.5	−0.2

* % Deviation is the difference in experimentally measured standard solution concentration with respect to that of nominal concentration expressed in percentage.

Table S7. Citrulline recovery study using QC samples.

Sample Text	Std. Conc. (ng/mL)	RT	Area	IS * Area	Response	Height	IS Height	Height/Area	S/N	Conc. (ng/mL)	% Deviation *
QC-1	7.5	1.34	283,29.67	120,122.1	0.236	427,982	1,824,037	15.107	367.705	9.2	22.5
QC-2	75	1.34	245,405.9	127,474.5	1.925	3,690,705	1,938,244	15.039	2339.951	79.2	5.6
QC-2	75	1.34	252,426.9	135,619.4	1.861	3,812,417	2,058,032	15.103	1643.134	76.2	2
QC-2	75	1.34	262,149.8	139,131.1	1.884	3,947,007	2,118,620	15.056	2047.953	77.5	3.3
QC-2	75	1.34	269,430.7	143,847.8	1.873	4,055,174	2,189,952	15.051	3303.92	77	2.7
QC-3	750	1.34	1,947,188	111,118.2	17.524	30,251,604	1,705,529	15.536	2381.648	725.4	–3.3

* IS stands for internal standard. * % Deviation is the difference in experimentally measured standard solution concentration and the nominal concentration expressed in percentage.

Table S8. Calcitroic acid recovery study using QC samples.

Sample Text	Type	Std. Conc. (ng/mL)	RT (min)	Area	Response	Height	Conc. (ng/mL)	% Deviation *
QC-1	QC	120	0.75	6845.377	6845.377	61058	109.2	–9
QC-2	QC	1200	0.74	78,885.273	78,885.273	655,633	1272.9	6.1
QC-2	QC	1200	0.74	78,180.914	78,180.914	645,773	1261.6	5.1
QC-2	QC	1200	0.74	72,488.844	72,488.844	594,802	1169.6	–2.5
QC-2	QC	1200	0.74	72,617.211	72,617.211	594,443	1171.7	–2.4
QC-3	QC	1800	0.73	105,384.79	105,384.789	866,361	1701	–5.5

* % Deviation is the difference in experimentally measured standard solution concentration and the nominal concentration expressed in percentage.

Table S9. Citrulline accuracy results.

Sr. No	Conc. Level *	Conc (ng/mL)	RT (min)	Area	IS Area	Response	Conc. (ng/mL)	% Deviation **	Average
1		1	1.08	5824.181	657.379	8.86	0.80	−20.0	
2		1	1.08	5806.071	643.988	9.016	1.10	10.0	
3	LLOQ	1	1.07	3111.904	346.241	8.988	1.10	10.0	−2.98
4		1	1.08	5246.309	584.452	8.976	1.00	0.0	
5		1	1.08	3582.166	402.461	8.901	0.90	−10.0	
6		3	1.07	4342.951	444.201	9.777	3.00	0.0	
7		3	1.08	4606.813	468.814	9.827	3.10	3.3	
8	Low QC	3	1.07	5005.619	518.435	9.655	2.70	−10.0	0.58
9		3	1.07	4259.939	433.799	9.82	3.10	3.3	
10		3	1.07	3947.511	396.971	9.944	3.40	13.3	
11		400	1.07	184,528.1	944.781	195.313	446.40	11.6	
12		400	1.07	177,638.5	962.83	184.496	420.50	5.1	
13	Middle QC	400	1.07	192,496	995.858	193.297	441.60	10.4	7.3
14		400	1.07	213,086.3	1095.628	194.488	444.40	11.1	
15		400	1.07	197,411.7	1140.784	173.049	393.20	−1.7	
16		800	1.07	354,614.7	978.262	362.495	846.00	5.7	
17		800	1.07	336,069.1	969.694	346.572	807.90	1	
18	High QC	800	1.07	313,254.4	910.373	344.095	802.00	0.3	2.42
19		800	1.07	329,387.8	877.368	375.427	876.90	9.6	
20		800	1.07	304,175	926.697	328.236	764.10	−4.5	

* LLOQ: Lower limit of quantification (1ng/mL); low QC: Lower quality control (3 ng/mL); medium QC: Medium quality control (400 ng/mL); high QC: High quality control (800 ng/mL); ** % Deviation is the difference in experimentally measured standard solution concentration and the nominal concentration expression in percentage.

Table S10. Within-run precision study for citrulline.

Sr. No.	Conc. Level *	Conc. (ng/mL)	RT (min)	Area	IS ** Area	Response	Conc. (ng/mL)	Standard Deviation	Mean	% CV ***
1		1	1.08	5824.181	657.379	8.86	0.8			
2		1	1.08	5806.071	643.988	9.016	1.1			
3	LLOQ	1	1.07	3111.904	346.241	8.988	1.1	0.12	0.98	11.9
4		1	1.08	5246.309	584.452	8.976	1			
5		1	1.08	3582.166	402.461	8.901	0.9			
6		3	1.07	4342.951	444.201	9.777	3			
7		3	1.08	4606.813	468.814	9.827	3.1			
8	Low QC	3	1.07	5005.619	518.435	9.655	2.7	0.22	3.06	7.3
9		3	1.07	4259.939	433.799	9.82	3.1			
10		3	1.07	3947.511	396.971	9.944	3.4			
11		400	1.07	184,528.1	944.781	195.313	446.4			
12		400	1.07	177,638.5	962.83	184.496	420.5			
13	Middle QC	400	1.07	192,496	995.858	193.297	441.6	20.26	429.22	4.7
14		400	1.07	213,086.3	1095.628	194.488	444.4			
15		400	1.07	197,411.7	1140.784	173.049	393.2			
16		800	1.07	354,614.7	978.262	362.495	846			
17		800	1.07	336,069.1	969.694	346.572	807.9			
18	High QC	800	1.07	313,254.4	910.373	344.095	802	38.75	819.38	4.7
19		800	1.07	329,387.8	877.368	375.427	876.9			
20		800	1.07	304,175	926.697	328.236	764.1			

* LLOQ: Lower limit of quantification (1ng/mL); low QC: Lower quality control (3 ng/mL); medium QC: Medium quality control (400 ng/mL); high QC: High quality control (800 ng/mL); ** IS stands for internal standard; *** CV stands for coefficient of variation.

Table S11. Inter-batch repeatability.

Sr. No.	Conc. Level*	Conc. (ng/mL)	Run-1 Conc.	Run-2 Conc.	Run-3 Conc.	Average Conc. (ng/mL)	Standard Deviation	Mean	% CV **
1	LLOQ	1	0.9	0.9	0.9	0.9	0.03	1	3.6
2		1	1	0.9	1	1			
3		1	0.9	1	1.1	1			
4		1	0.9	0.9	1.1	1			
5		1	0.9	1	0.9	0.9			
6	Low QC	3	2.9	3.4	3	3.1	0.1	3.1	3.4
7		3	3	3.3	3.1	3.1			
8		3	2.8	3.3	2.7	2.9			
9		3	3.4	2.9	3.4	3.2			
10		3	3	3.3	2.7	3			
11	Middle QC	400	458	447.6	433.5	446.4	12.38	430.3	2.9
12		400	448.8	419.5	453.3	440.5			
13		400	439.7	439.9	411.8	430.5			
14		400	447.3	417.1	403.2	422.5			
15		400	455	401.5	378.8	411.8			
16	High QC	800	881.2	737.4	712.5	777	28.29	815.8	3.5
17		800	877.1	827.4	710.8	805.1			
18		800	881.8	756.8	799.9	812.8			
19		800	889.9	746.8	822.2	819.6			
20		800	893.1	825.4	874.7	864.4			

* LLOQ: Lower limit of quantification (1 ng/mL); low QC: Lower quality control (3 ng/mL); medium QC: Medium quality control (400 ng/mL); high QC: High quality control (800 ng/mL); ** CV stands for coefficient of variation.

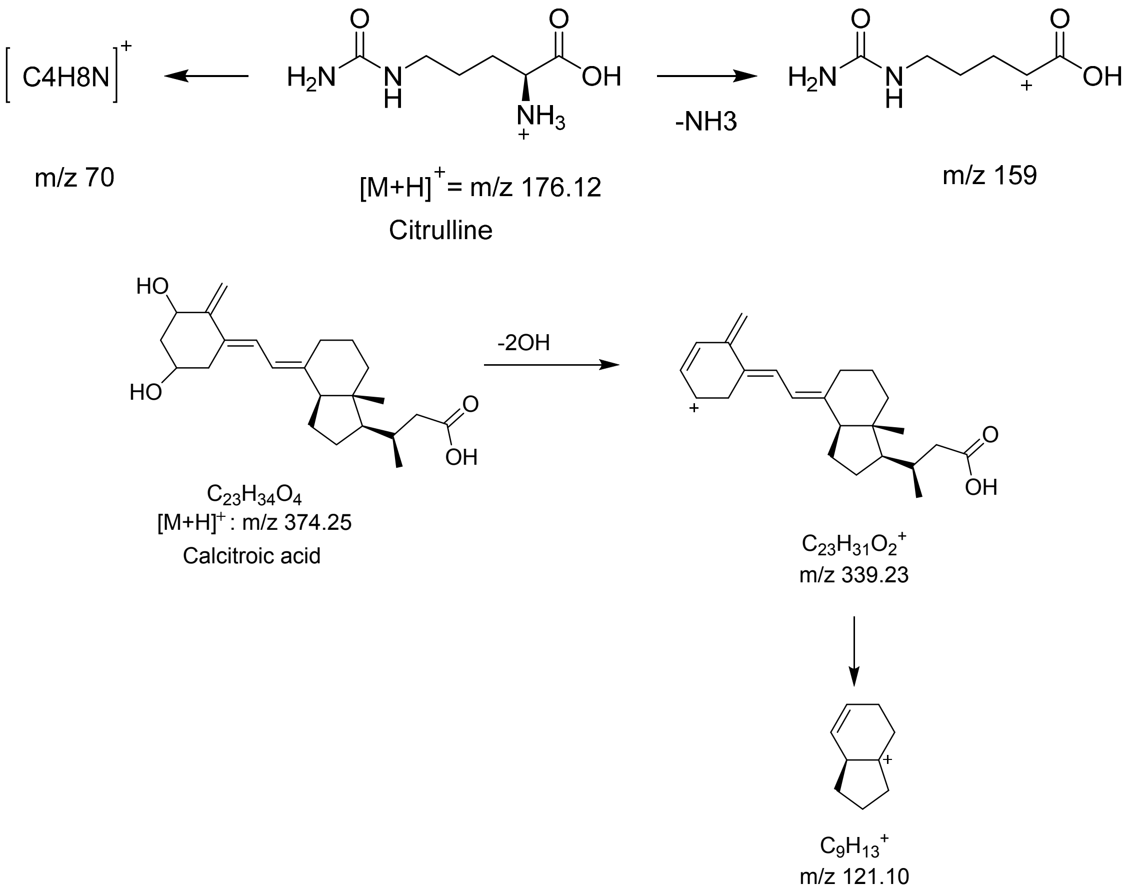

Figure S1. Fragment structures for citrulline and calcitroic acid transitions.

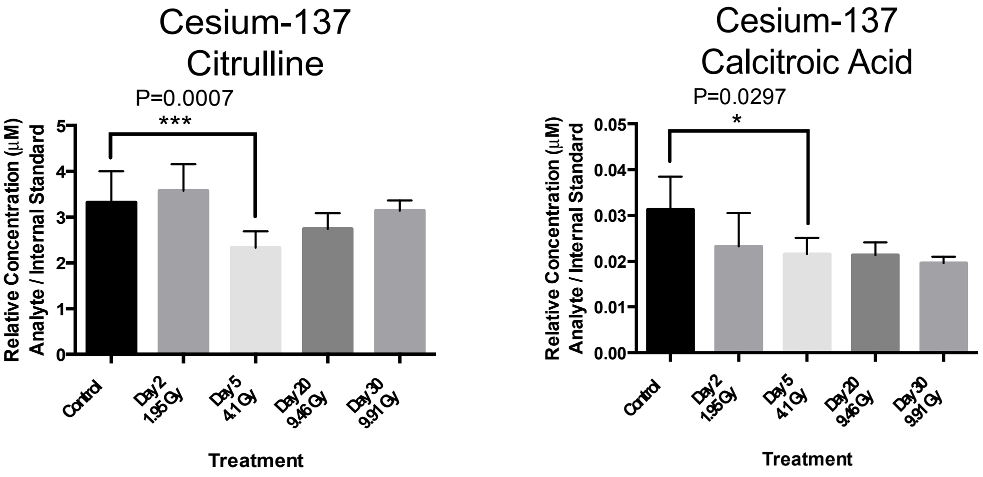

Figure S2. Decrease in urinary excretion of citrulline and calcitoic acid after internal exposure to Cesium-137 at different time-points in a 30-day study. (***) denotes statistical significance of the change in urinary excretion 0f citrulline in terms of *p*-value (p = 0.0007) while (*) denotes that of calcitroic acid (p = 0.0297) 5 days after the exposure.

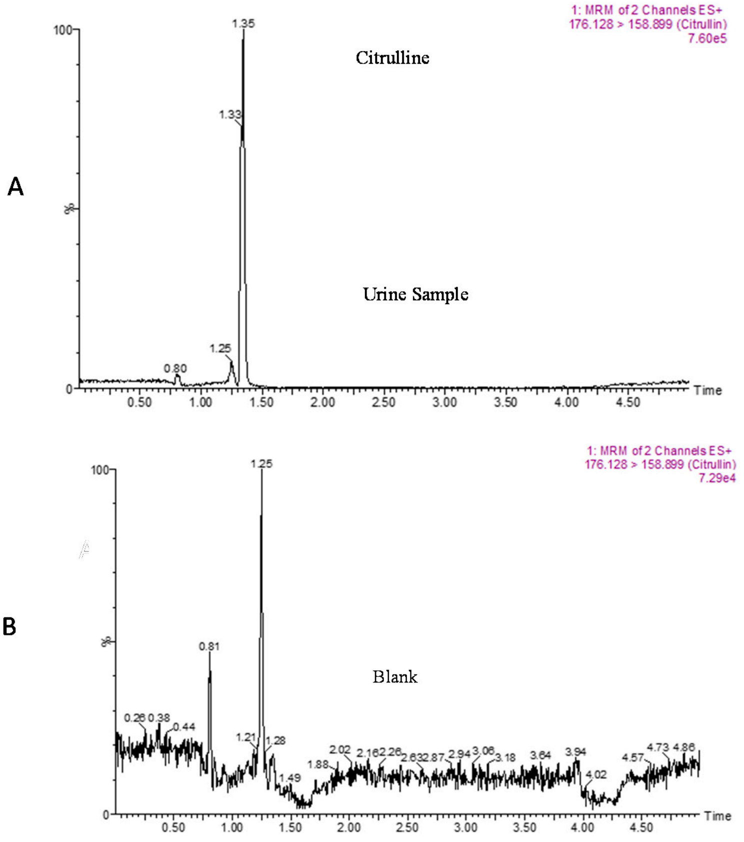

Figure S3. Test (**A**) and blank (**B**) chromatograms for citrulline.

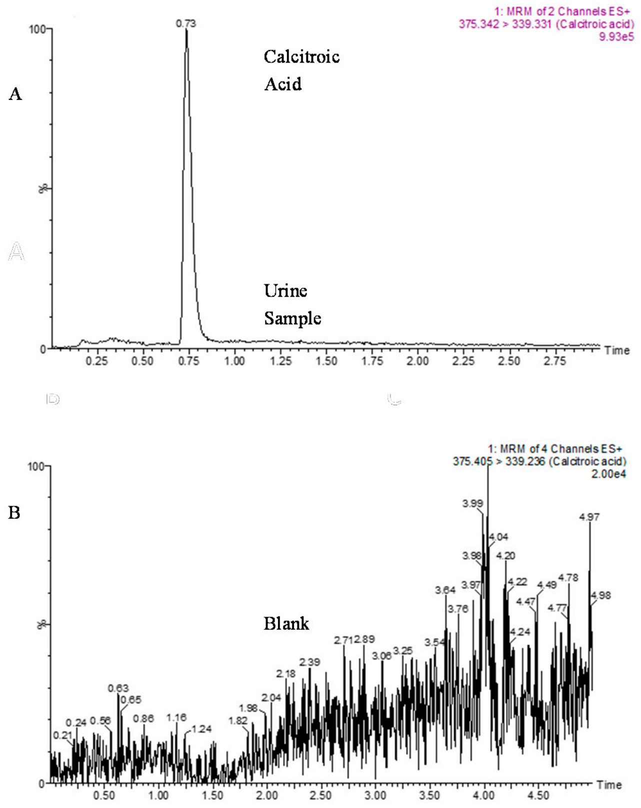

Figure S4. Test (**A**) and blank (**B**) chromatograms for calcitroic acid.

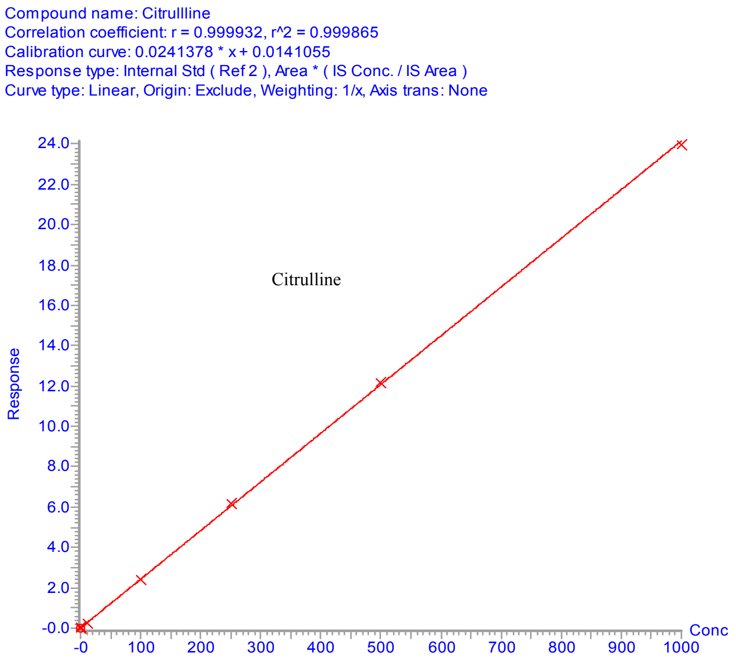

Figure S5. The linear concentration range of the calibration curve for citrulline ranging from ng/mL to 1000 ng/mL.

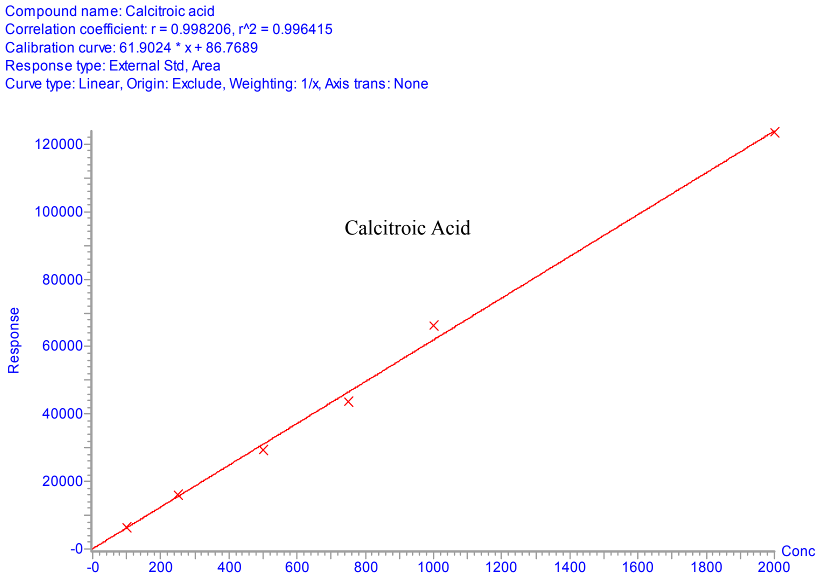

Figure S6. The linear concentration range of the calibration curve for calcitroic acid ranging from 100 to 2000 ng/mL.
